# Examining the International Palliative Care Systems in Rural Areas: Protocol for a Comparative Case Study

**DOI:** 10.2196/36037

**Published:** 2022-07-01

**Authors:** Meritxell Mondejar-Pont, Kristen Abbott-Anderson, Anna Ramon-Aribau, Renee Kumpula, Tammy Neiman, Hans-Peter De Ruiter

**Affiliations:** 1 Faculty of Health Sciences and Wellness University of Vic Vic Spain; 2 School of Nursing Minnesota State University Mankato, MN United States

**Keywords:** palliative care, palliative care systems, integrated palliative care, global health comparison, hospice, rural health, ethical dilemmas, COVID-19, coronavirus, complementary therapy

## Abstract

**Background:**

The aging population in the Global North is associated with an increased prevalence of multiple chronic diseases that would benefit from integrated palliative care. In this context, it is vital to consider the effectiveness of health care systems’ response to the needs of the older population residing in rural areas, including access to palliative care services. Understanding palliative care program availability and palliative care system characteristics is important in creating useful health interventions in rural areas.

**Objective:**

This study aims to provide an international view on palliative care in rural areas. A study exploring palliative care services offered in Southern Minnesota will be carried out, building on a previous study conducted in Osona, Spain. Findings from both studies will be compared, providing insights into the strengths of each system and identifying areas for growth.

**Methods:**

This study will be performed using qualitative case study methodology. Using a similar methodology to the one used in the Spanish study, palliative care services will be explored in a similarly sized rural area in Southern Minnesota. This will be accomplished by (1) reviewing available literature related to the Southern Minnesota palliative care system and (2) identifying key providers in this US palliative care system who will be invited to participate in semistructured interviews. The study participants will be asked about the gaps between ideal integrated palliative care system services and the existing complementary palliative care services, and the ethical issues and dilemmas that evolved during the COVID-19 pandemic.

**Results:**

Following ethical approval for this protocol, data collection is anticipated to begin in spring or summer 2022 and is expected to take 6 months. Data collection will be followed by data analysis in fall 2022. Finally, the researchers plan to disseminate the findings in spring 2023.

**Conclusions:**

Comparing 2 similarly sized but culturally different rural palliative care systems in Minnesota and Osona will provide insights into how integrated palliative care systems impact the older population and those with chronic illnesses. Study findings will contribute to enhanced patient care, organizational improvements, policy change, and an understanding of the impact of different health care system models.

**International Registered Report Identifier (IRRID):**

PRR1-10.2196/36037

## Introduction

An aging population in the Global North is associated with an increased prevalence of chronic diseases that will ultimately contribute to their death [1]. Due to the population’s increasing life span and resultant aging, it is essential to consider how effectively health care systems, especially palliative care services, respond to patients with chronic illnesses [2-4].

The World Health Organization states that palliative care aims to improve quality of life for those patients living with life-limiting diseases, by reducing pain and proactively managing and treating symptoms associated with their disease processes [5]. Historically, palliative care was limited to patients with cancer [6] and was described as care for individuals with a life-threatening disease [1]. With an aging population, palliative care systems evolved and are no longer limited to oncological or life-threatening diseases [7]. Palliative care currently includes patients with multiple morbidities [8] and individuals in need of quality-of-life–focused care due to frailty, advanced age [9], or disabling conditions [10,11]. Thus, palliative care services are justified based on individual patients’ needs rather than specific diagnostic codes. The target patient for palliative care is an individual with a “palliative cluster” of symptoms or factors, including a life-threatening or life-limiting, chronic, or terminal condition, necessitating multidimensional needs.

Past health care models focused on disease-directed care in which the recognition of the terminal nature of a patient’s condition occurs late, resulting in delayed hospice and end-of-life care [12]. However, the changes in managing chronic and life-limiting conditions to enhance the quality of life have resulted in a new model in which palliative care service is initiated at the time of diagnosis. In this model, the focus shifts toward symptom management rather than cure, and a transition to hospice care when a patient’s life expectancy is 6 months or less; during this time, the primary purpose of care is maximizing the patient’s quality of life [13].

A challenge for health care systems is how to provide integrated care for patients with increasingly complex chronic conditions [14]. Integrated care is a combination of multiple disciplines at different levels of care, focused on improving the quality of health care services [3]. It also facilitates connections and cooperation between funding sources, organizations, and clinical services, with the purpose of offering efficient and high-quality care. As a result, integrated palliative care (IPC) provides coordinated services of care [15].

For patients in Southern Minnesota, access to palliative and hospice care services can differ based on the type of insurance the patient has. For example, the national insurance for the older population, Medicare Part A, covers inpatient hospital stays, short-term stays at nursing facilities, and home care [16]. Reimbursement for palliative care is not standardized as it is for hospice care [17,18]. In contrast, the Spanish health care system provides universal access to all residents. Instituto Nacional de la Salud (the government’s public health organization) provides health care services to all communities in the country [19], and these services include palliative care for patients with chronic illnesses and terminal diseases.

In both countries, with aging populations, the main challenge for health care systems is to provide integrated care for patients with increasingly complex chronic conditions [14]. IPC is an approach to improve services for patients with chronic illnesses and terminal diseases.

The COVID-19 pandemic can be a distinguishing marker between the period considered “normal” and the pandemic era, which brought many changes to society and health care delivery [20,21]. The pandemic also impacted health care providers who were faced with ethical dilemmas such as determining resource allocation and the prioritization of patient care [22], as well as end-of-life care decisions [23]. Additional ethical concerns encountered by palliative care providers were autonomy in the patient and family decision-making processes [24], the discontinuation of treatments and therapies [23], and communicating with families and patients in a society that required social distancing and limiting factors such as face masks [22].

When taking care of patients with chronic conditions, integrative and complementary care is often experienced as beneficial [25]. The integrative health approach provides patients in need of palliative care with nonpharmacological strategies to manage pain and other nausea, depression, and anxiety symptoms [26]. Examples include aromatherapy, acupuncture, massage, homeopathic practices, and cultural practices [27]. In addition, palliative care in conjunction with complementary care can offer patients comfort during this phase of life [26].

One way to see how care is being delivered to people with complex chronic conditions is through the analysis of reality; therefore, this study aims to first describe the Southern Minnesota palliative care system in the United States and then compare it to the palliative care system in Spain. To be able to carry out the general objective of the study, the following specific objectives will be developed:

1. Describing the palliative care system in Southern Minnesota
2. Comprehending the ethical dilemmas health care providers encounter while providing care in the Southern Minnesota palliative care system
3. Identifying specific impacts resulting from the COVID-19 pandemic
4. Assessing the complementary services offered by palliative care service providers in Southern Minnesota
5. Identifying and comparing the commonalities and differences between this study in Southern Minnesota and the results found in a previous study from Osona, Spain

## Methods

### Design and Methods

This research will follow the same design and methods used in the study conducted in Osona, Spain (M Mondejar-Pont, PhD, unpublished data, November 2020). This study will use a qualitative methodology with a prospective, multiple embedded case study design as described by Yin [28]. This design allows us to explore the embedded subunits of multiple cases to understand more about the case itself.

This study will describe the Southern Minnesota palliative care service and its essential integrated palliative care system elements, identify the ethical dilemmas experienced, and identify the complementary therapies offered. The results found in this study will then be compared to the results found in the initial study conducted in Osona, Spain. This comparison will aim to identify similarities, differences, and informative aspects that may benefit each system, while taking into consideration the contextual and cultural differences between the two.

### Case Selection

Blue Earth, Nicollet, and Brown counties in Southern Minnesota were selected for this study based on similarities between these areas and the region in Spain that is the population of comparison. In addition, Blue Earth, Nicollet, and Brown Counties include the Mankato metropolitan area and the surrounding rural areas [29] that have significantly smaller populations and less access to health care services. These counties will be referred to as Southern Minnesota for ease of readability.

These 2 regions were selected since they have similar populations: Osona county has a population of 163,702 [30], and Southern Minnesota has a population of 125,912 [31]. In these regions, the older population is represented with a similar proportion: 18% in Osona [32] and 24% in Southern Minnesota [33]. With a significant proportion of rural populations aging in place, access to palliative care service is imperative yet more challenging outside of larger urban areas [34]. By comparing and contrasting two similarly sized regions with different health care systems and reimbursement models, this research will ultimately provide information that the palliative care systems in both regions can use to improve their practices.

### Participants

Consistent with the study completed in Osona, Spain, this study will use a purposive sampling strategy including the following 2 types of participants who will be invited to take part in the study: (1) those who hold decision-making positions in organizations providing palliative care, including managers, coordinators, or lead administrators; and (2) professionals involved in the provision of palliative care, such as nurses, social workers, and physicians.

We anticipate interviewing up to 25 participants, similar to the study completed in Spain, representing a wide variety of roles within palliative care systems. Interviews will be analyzed using the direct content analysis approach explained further below, and the analysis will conclude once the research team determines that data saturation has been reached. The research team will determine that data saturation has been reached when no new additional information, new codes, or categories are possible to obtain. If data saturation is not reached after 25 interviews, interviews will continue until saturation is reached.

Initially, professionals in leadership positions will be interviewed about the palliative care system. The interviews with individuals in leadership positions aim to gain a holistic sense of the organization, communication, and coordination efforts at the macro level. These professionals, following a snowball strategy, will provide contact information of direct care providers. Direct care providers can give more detailed insights about the palliative care system at the micro level.

### Data Collection

The study will be divided into the following 2 phases to respond to the study’s main goals:

- Phase 1 aims to identify a description of the palliative care system in Southern Minnesota through a search in the available documents and literature review.
- In Phase 2, the aim is to identify the integrated elements of palliative care systems; the ethical dilemmas encountered prior to, during, and in the current phase of the COVID-19 pandemic; and the complementary care offered. During this phase, key personnel and direct health care professionals will be interviewed individually by members of the research team. These semistructured interviews will take place via teleconferencing or phone. All interviews will be audio recorded (Multimedia Appendix 1 includes the survey questions) to be later transcribed and analyzed. Finally, the results from this study of the Southern Minnesota palliative care system will be compared with the results found in the research completed in Osona, Spain.

### Data Management and Analysis

Anonymous participant data will be stored in a protected database such as Microsoft Teams with a login function. The master database will be kept in a password-protected, university-issued computer.

Interviews will be audio recorded and transcribed verbatim. Transcriptions will be analyzed using deductive content analysis supported by the qualitative data analysis software NVivo (version 12; QSR International).

The deductive or directed content analysis approach uses previous research findings to examine the studies’ new data to identify similarities and differences and compare the same categories at different times and in other locations. Deductive content analysis has 3 main processes: data preparation, organization, and reporting. In the data preparation phase, a matrix of categories from existing theories is created and then compared to the emerging categories from the study’s data [35]. In the organization phase, documents and interviews will be analyzed using deductive content analysis, and prior theoretical propositions will guide the initial coding process and formation of first categories. Then, new categories emerging from the data will be generated, and finally, links between initial and newly generated categories will be established and reported as results.

The research team members will individually review study findings, identifying themes, concepts, and case components; the research team will then meet to discuss results until consensus and data saturation are reached. Once themes, concepts, and case components have been identified for the Southern Minnesota palliative care system, they will be compared to those in Osona’s palliative care system. Similarities and differences will be used to identify system strengths and areas for growth.

### Ethical Review

Informed consent will be obtained to assure voluntary participation. Participants may withdraw at any time at their discretion. Therefore, we believe that the potential for risk in this study is minimal.

To minimize the burden of data collection on busy professionals, interviews will be limited to a maximum of 60 minutes. Interviews will be conducted by experienced researchers.

An application for ethical approval has been submitted to the Institutional Review Board of Minnesota State University, Mankato and is awaiting approval (1877595).

## Results

This study was initiated in August 2021, when the research team met and established its organization and the project goals. The Institutional Review Board application was submitted in April 2022, and further project planning has been undertaken during spring 2022. Results are pending ethical review and data collection, which will take place in spring and summer 2022, followed by data analysis in fall 2022. Dissemination of results and development of various study reports will be anticipated after data analysis is completed in 2023.

Anticipated results for this study are expected to be consistent with those found in the foundational study completed in Osona, Spain. In that study, major themes included a need for improved collaboration, continuity of care, and sustainable funding. Ethical dilemmas identified included the decision to continue nonbeneficial treatment, life-sustaining and life-prolonging therapies, and palliative sedation.

## Discussion

Anticipated outcomes for this study on IPC in Southern Minnesota will include suggestions to enhance patient care, improve organizational structures, and change policy, as indicated by the study findings. Understanding ethical dilemmas encountered by palliative care service providers and the complementary therapies used will identify new patient-centered care strategies.

There is currently an increased interest in IPC, an optimal approach to provide care for patients with chronic conditions and terminal illnesses [36]. However, the literature indicates that there is no agreement on the definition of IPC’s essential components, and thus, there is the need to define its integral elements [37,38]. Consequently, IPC implementation varies across settings, and understanding its application is complicated. Describing and comparing different IPC systems such as the ones in Southern Minnesota and Osona offers greater insights into the implementation of IPC systems in 2 different countries.

The COVID-19 pandemic offers an opportunity for reflection and a new interpretation of health issues, especially in the ethical domain [20], such as resource provision and care prioritization [22]. In addition, the pandemic has exposed unique health-related ethical dilemmas [39], resulting in more complex decision-making processes [23]. This study will reveal the ethical dilemmas Southern Minnesota palliative care providers have encountered during the COVID-19 pandemic and compare them to those confronted by health care professionals in Osona’s palliative care system.

In summary, further research on the implementation and evaluation of IPC systems is needed. Describing the essential elements, ethical dilemmas, and complementary therapies of the Southern Minnesota palliative care system will bring a greater understanding of their implementation within the IPC systems. Additionally, comparing 2 IPC systems that are similar in population and rural setting will provide a richer understanding of the impact of IPC systems on people with chronic illnesses. Study findings will contribute to enhanced patient care, organizational improvements, policy change, and a better understanding of the impact of different health care system models.

## Appendix

Multimedia Appendix 1 Interview script.

## Abbreviations

IPC: integrated palliative care
