# Supplementary material for: Examining the International Palliative Care Systems in Rural Areas: Protocol for a Comparative Case Study
Source: JMIR Res Protoc. 2022 Jul 1;11(7):e36037. doi: 10.2196/36037 (PMC9288105; doi:10.2196/36037)
Supplement: Multimedia Appendix 1 [file resprot_v11i7e36037_app1.docx]

**Appendix A.** *Interview Script*

**Interview**

Palliative cares system of Southern Minnesota

**1**. Imagine a perfect or ideal model of an integrated palliative care system (PCS) for the advanced chronically ill and end-of-life patients. What elements should it have? (warm-up question)

Please specify (within this perfect/ideal model of an integrated palliative care system (IPCS)? for the chronically ill and end-of-life patient) what these factors would be / how they would function? (if not stated above):

- professionals providing palliative services in an integrated system.
- collaboration within the system of an integrated approach.
- sharing information within an integrated system.
- patient detection
- organization in the system as an integrated system.
- the services offered to palliative patients

2. Now, I would like you to think about the Southern Minnesota PCS. Comparing the MPCS with an Ideal Integrated Palliative Care System, what factors/elements does the Southern Minnesota PCS have as an integrated palliative care system for chronically ill and end-of-life patients?

3. What factors/elements mentioned above are missing or lacking in the Southern Minnesota PCS? Why don't we have them?

4. In your professional practice, what kind of ethical dilemmas (complicated situations as a professional) do you find yourself in when providing integrated services to chronically ill and end-of-life patients?

**5.** What ethical dilemmas do you think health personnel has encountered due to the COVID-19 pandemic with the chronically ill and end-of-life patient?

6. Are there any complementary/integrative therapies or services provided to the end-of-life or palliative patients in Southern Minnesota? Can you tell me which ones?
